# Editorial: Synthetic biology in Vertebrate Model system: New clinical and preclinical translational applications

**DOI:** 10.3389/fmolb.2023.1117896

**Published:** 2023-02-03

**Authors:** Xu Wang

**Affiliations:** ^1^ Cancer Research Institute, Pancreatic Cancer Institute, Fudan University Shanghai Cancer Center, Shanghai, China; ^2^ Shanghai Pancreatic Cancer Institute, Shanghai Key Laboratory of Radiation Oncology, Fudan University, Shanghai, China

**Keywords:** synthetic biology, vertebrate model, biology circuit, non-biological structure, cell therapy

Synthetic biology represented the conceptual and technological merging of life science with engineering and information science, with the goal of enabling scientists to rationally create, design, and manipulate life with programmable functions. For more than half a century, the typical chassis cells of synthetic biology were *E. coli* and *S. cerevisiae*, and many synthetic circuits have been developed and poised to transform those cells into productive tools in the biotechnology and medicine industry (Cameron et al., 2014). However, the field of synthetic biology has expanded rapidly in the past decade (Meng and Ellis, 2020), and vertebrate cells including human cells have become novel platforms for integrating synthetic technologies, including genome editing (Shalem et al., 2014), light/chemical-sensing circuitry (Deisseroth, 2011), ligand/antigen-controlled circuitry (Morsut et al., 2016; Melenhorst et al., 2022), multi-unit transgenesis (Cai et al., 2013), *etc.* As time goes on, the current boarder of synthetic biology field becomes vague, and synthetic technologies are present across multiple aspects of biomedical research.

Here we gathered several experts in synthetic biology as the editorial group, Wang Yongming’s laboratory was dedicated to the optimization of gene editing technologies, and have developed several novel tools including SlugCas9 (Hu et al., 2021) and SchCas9 (Wang et al., 2022) for potential applications in gene therapy. Zhao Yuzhen’s laboratory has developed several artificial and genetically encoded fluorescent sensors, including NAD+/NADH sensors, to reveal metabolic dynamics in living model organisms (Zhao et al., 2015; Zou et al., 2020). In my laboratory, we have generated several transgenic vertebrate cancer models using synthetic sequences, which were composed of multiple regulatory units like “LeGo” blocks (Yao et al., 2018; Fei et al., 2019; Fei et al., 2021). We also employed ligand-controlled synNotch receptor and antigen-recognizing CAR receptor to engineer human T cells, which were programmed to attack human cancer cells or animal cells expressing human cancer antigens (Wang et al., 2021). In addition, we designed double-humanized animal models carrying semi-immortalized primary xenografts, and aligned the models in three-dimensional printed chips, followed by automatic drug screening and high-throughput imaging analysis, as a proof of concept combining both synthetic animal models and customer-made abiotic structures (Wu et al., 2023). Together, these experiences formed the original idea for this topic, in which we hoped to include a diversity of synthetic elements from different research backgrounds.

In the research topic, Wang Zhu’s laboratory reviewed their progress in using multi-target CRISPR/Cas9 technology to generate animal models of prostatitis and prostate cancer (PCa), and employing synthetic circuits to genetically trace the cell-of-origin of PCa (Bleeker et al.), representing the application of synthetic cellular/genetic circuits in vertebrate systems. Another work combined GBM cell culture with biocompatible elastic materials to build a synthetic encephaloid model for brain stiffness mimicking and drug screening, representing the contribution of abiotic materials to the engineering and modeling of complex biological systems (Wang et al.). Beyond this topic, other researchers may pick up these synthetic blocks and make their own synthetic models for different purposes and applications.

In general, engineering of synthetic biology in vertebrate systems remains challenging, including multiple levels of regulation, fluid cell fate determination, and complex multicellular interactions. Nevertheless, the current elements of synthetic biology have formed a foundation or library for studying the spatiotemporal dynamics of diseases and for developing relevant diagnostic or therapeutic approaches, and we are looking forward to much more significant progress in their clinical and preclinical translational applications.
